# Informed consent procedure in a double blind randomized anthelminthic trial on Pemba Island, Tanzania: do pamphlet and information session increase caregivers knowledge?

**DOI:** 10.1186/s12910-019-0441-3

**Published:** 2020-01-06

**Authors:** Marta S. Palmeirim, Amanda Ross, Brigit Obrist, Ulfat A. Mohammed, Shaali M. Ame, Said M. Ali, Jennifer Keiser

**Affiliations:** 1 Swiss Tropical and Public Health Institute, Basel, Switzerland; 2 University of Basel, Basel, Switzerland; 3 Public Health Laboratory Ivo de Carneri, Chake Chake, Tanzania

**Keywords:** Informed consent, Understanding, Information session, Pamphlet, Clinical trial, Hookworm, Mebendazole

## Abstract

**Background:**

In clinical research, obtaining informed consent from participants is an ethical and legal requirement. Conveying the information concerning the study can be done using multiple methods yet this step commonly relies exclusively on the informed consent form alone. While this is legal, it does not ensure the participant’s true comprehension. New effective methods of conveying consent information should be tested. In this study we compared the effect of different methods on the knowledge of caregivers of participants of a clinical trial on Pemba Island, Tanzania.

**Methods:**

A total of 254 caregivers were assigned to receive (i) a pamphlet (*n* = 63), (ii) an oral information session (*n* = 62) or (iii) a pamphlet and an oral information session (*n* = 64) about the clinical trial procedures, their rights, benefits and potential risks. Their post-intervention knowledge was assessed using a questionnaire. One group of caregivers had not received any information when they were interviewed (*n* = 65).

**Results:**

In contrast to the pamphlet, attending an information session significantly increased caregivers’ knowledge for some of the questions. Most of these questions were either related to the parasite (hookworm) or to the trial design (study procedures).

**Conclusions:**

In conclusion, within our trial on Pemba Island, a pamphlet was found to not be a good form of conveying clinical trial information while an oral information session improved knowledge. Not all caregivers attending an information session responded correctly to all questions; therefore, better forms of communicating information need to be found to achieve a truly informed consent.

## Background

Informed consent is an essential ethical and legal requirement in research involving human subjects [1]. Nowadays, it is expected that clinical trials adhere to the International Conference on Harmonisation Good Clinical Practice (ICH GCP) guidelines [2]. These guidelines define informed consent as “a process by which a subject voluntarily confirms his or her willingness to participate in a particular trial, after having been informed of all aspects of the trial that are relevant to the subject’s decision to participate” and the 9th principal of ICH GCP is that “freely given informed consent should be obtained from every subject prior to clinical trial participation” [2].

Proof of obtaining informed consent is documented using a written, signed and dated informed consent form (ICF) [2]. Informed consent is a procedure through which subjects, after having received the entire content and procedures of the clinical trial, can voluntarily provide willingness for participation; it is the investigator’s responsibility to ensure that the consent process is conducted appropriately [2, 3]. Although the GCP guidelines provide a list of topics which should be presented both in the informed consent discussion and in the ICF, they do not give any advice on how to provide this information. Different methods and educational materials can be used to properly inform subjects, but the selection of the techniques is entirely in the hands of the researchers themselves, leaving room for uninformed decisions or even misconduct. Many trials still rely on lengthy and complex ICFs alone to transfer all relevant information to participants [4]. For decades it has been emphasized that the traditional ICF, in most cases, is not a sufficiently good method of conveying information since often participants do not read the ICF [5] or fail to understand after reading it [6, 7]. The fact that many investigators view ICFs as mere legal proof of a participant’s agreement has increased their complexity, and consequently reduced the participant’s ability to understand its content. This results in an informed consent process, which is legally correct, but does not guarantee the participant’s true comprehension of the study [8]. People often confuse receiving medical care with participating in a clinical trial [9, 10], or do not understand concepts such as randomization, the right to withdraw at any time, and the risks and benefits of participation [11]. More visual, interactive and engaging forms of conveying information are required where an ICF does not truly inform.

Additionally, in practice, researchers usually do not know to what extent individuals have understood the aim, procedures, their rights, benefits and potential risks of a clinical trial when they agree to participate [12, 13]. This issue is particularly alarming in low- and middle-income countries where a combination of low educational levels, poor access to health care and low health literacy levels increase the risk of uninformed consenting. Indeed, it has been shown that participants in low- and middle-income countries are less likely to refuse to participate in clinical research as well as withdraw from it when compared to participants in developed countries [1, 14]. Moreover, the information which a subject uses to decide whether or not to participate in a clinical trial, is often communicated inappropriately in limited-resource settings [8].

The present study aimed at assessing whether diverse forms of delivering the same informed consent information generated different levels of understanding in caregivers of clinical trial participants receiving two anthelminthic treatment regimens on Pemba Island, Tanzania. Using a short questionnaire, we compared the clinical trial-related knowledge of caregivers assigned to groups with different forms of providing information: (i) pamphlet only, (ii) oral information session only, and (iii) pamphlet plus oral information session. A forth group of caregivers served as control and did not receive any information other than the ICF before the interview (an information session was conducted after completion of questionnaire).

## Methods

### Study design, ethics and participants

The study was embedded in a school-based, randomized, double-blinded clinical trial conducted at Piki Primary School, on Pemba Island, Tanzania, from July to September 2017. The primary objective of the trial was to evaluate the safety and efficacy of a multiple dose mebendazole regimen (3 days 100 mg bid) versus a single dose of 500 mg mebendazole against hookworm infections in 186 children aged 6 to 12 years. The methodology and details of this clinical trial (number NCT03245398, ClinicalTrials.gov) have been published elsewhere [15]. Caregivers, who allowed the participation of their child, were asked to sign a written informed consent. Illiterate caregivers provided a thumbprint while an impartial witness signed to verify that all information in the informed consent form was conveyed correctly.

### Information session and pamphlet

All children invited to participate in the clinical trial were orally informed about the date and time of the information session, which one of their caregivers should attend. Children were given an ICF, which they should hand over to their caregiver before the information session. The information session covered all important topics included in the ICF in the most simple and clear language possible. The content and language of the speech were discussed together with the local research staff and were standardized (see Additional file 1). Caregivers were encouraged to ask any questions they may have before deciding whether their child should participate in the trial or not.

**Fig. 1:**
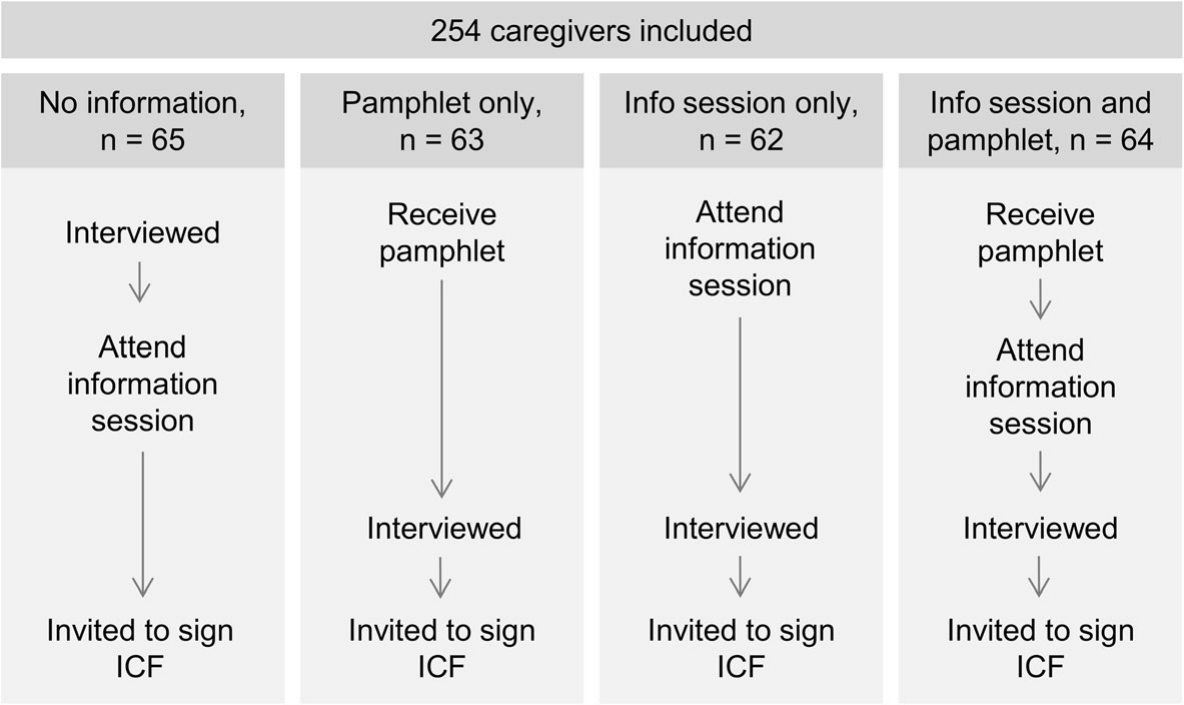
Flow diagram of the order of procedures in each of the four groups of caregivers

Upon the announcement of the date and time of the information session, the research staff handed out to half the children a pamphlet addressed to their caregivers, which was developed and adapted to the local culture and conditions by the research team and local staff (Additional file 2). Figure 1 describes the study flow in each of the caregiver groups.

The key messages of the information session and the pamphlet were the same and included the following information: (i) hookworm is a parasite, which infects us through the skin when we walk barefoot; (ii) hookworm can cause a child to not develop properly and have difficulties at school; (iii) one can treat hookworm with a specific medication; (iv) in this study we intend to find the “best amount” of mebendazole to cure their child; (v) mebendazole can cause mild adverse events; (vi) the caregiver should be the one deciding whether the child participates or not; (vii) if the child remains infected with hookworm after treatment he/she will receive another drug (tablet); (viii) the child can withdraw from the study at any time, with no consequence and would still receive treatment; (ix) the treatment is free, nobody is paid to participate but all caregivers would be reimbursed USD 2 to cover their transport to the information session; (x) only the child’s caregivers and research staff will have access to his/her personal information; (xi) to participate each child will have to provide several stool samples and a small blood sample but no urine sample; and (xii) children should inform the study team about how he/she is feeling after treatment and allow a doctor to check his/her overall health.

### Questionnaire and data collection

Data were collected using a questionnaire consisting of ten multiple choice questions (each with four choices) and seven true or false questions (Table 1, Additional file 3). The questionnaire was orally administered in Swahili by six trained interviewers. Only one caregiver per child was interviewed. Caregivers who had more than one child invited to participate in the trial were only interviewed once. After being interviewed, caregivers in the control group attended an information session before they were invited to sign the ICF. Caregivers who chose not to sign the ICF for their child to participate in the clinical trial but still agreed to respond to the questionnaire were included in this study. Interviewers visually estimated the caregivers’ age and recorded it. Children of caregivers participating in this survey also responded to several socioeconomic questions inquiring about possession of specific household assets (soap, radio, television, computer, cell phone, fridge, fan, bike, scooter, car, tractor, electricity) and household conditions (source of drinking water, type of roof, walls and floor).

**Table 1:** List of multiple choice and true/false questions. Correct multiple choice answers are highlighted with an arrow and true/false questions are marked with T (true) or F (false)

Multiple choice questions		
1. What is hookworm?		
	A	A worm that can infect us if we drink dirty water.
→	B	A worm that can go into our feet if we walk barefoot.
	C	A worm that can infect us if we eat rotten food.
	D	A worm that can get to our food through flies.
	E	Don’t know.
2. Why is hookworm bad for your child?		
	A	My child will get pimples all over the body.
	B	My child’s urine will become red (blood).
→	C	My child may not grow well and may have difficulties at school.
	D	My child will be very hungry all the time.
	e	Don’t know.
3. Is it possible to treat hookworm?		
	a	No, it is not possible to treat hookworm.
	b	Yes, it is possible to treat if my child eats a lot of healthy food like veg/fruit.
	c	Yes, I should take my child to the traditional healer.
→	d	Yes, my child can receive medication that will kill the hookworm.
	e	Don’t know.
4. What do we want to do in this study?		
	a	To see if mebendazole kills the worms in my child’s belly because this drug has never been used before.
	b	To see if mebendazole kills the worms that are in my child’s feet.
	c	We want to see if mebendazole is better than another drug called albendazole at killing the worms.
→	d	We want to find out what is the best amount of a mebendazole to kill the hookworm in my child’s belly.
	e	Don’t know.
5. Who should decide if your child should participate in this study?		
	a	Teacher
→	b	Mother/father
	c	Doctors or nurses
	d	Neighbor or relative
	e	Don’t know.
6. What happens if your child still has worms after the treatment?		
	a	There is nothing that we can do
	b	We will give him/her an injection to kill the worms
	c	Your child should drink a lot of water
→	d	We will give him/her another pill to kill the worms
	e	Don’t know.
7. Can your child give up participating during the study?		
→	a	Yes, my child can give up and there is no consequence. He/she will still receive treatment.
	b	Yes, but my child will not receive treatment.
	c	No, my child cannot give up if I decide he participates he/she has to stay until the end of the study.
	d	Only if the doctor and teacher agree that he/she can give up.
	e	Don’t know.
8. What about payment?		
	a	There are some costs for you: you will have to pay for your child’s treatment.
→	b	There are no costs for you: the treatment is free and you will get 2$ if you came to the information session.
	c	You will receive money if your child accepts the treatment
	d	You will only get money if the treatment kills the worms
	e	Don’t know.
9. Is the treatment we will give to your child (mebendazole) safe?		
	a	Nothing bad can happen if my child takes the treatment.
	b	If my child takes the treatment he/she will not be able to walk for a few days.
→	c	If my child takes the treatment, he/she may feel some things like a belly ache or a headache but nothing very dangerous.
	d	If my child takes the treatment he will sleep all day.
	e	Don’t know.
10. Who will be able to see your child’s personal information?		
	a	Neighbors
→	b	Only study investigators
	c	Teacher
	d	Only you
	e	Don’t know.
True or false questions		
11. What does your child need to do if he/she participates in this study? Will he have to …		
T	a	Give us several stool samples?
F	b	Pay for the medication to kill the worms (mebendazole)?
T	c	Tell us if he/she is feeling well after the medication?
F	d	Give us a urine sample?
F	e	Only give us one stool sample?
T	f	Give us a small blood sample?
T	g	Accept that a doctor checks his/her health?

Initially, the questionnaire was developed in English by the authors. A meeting including the five local interviewers with the aim of adapting the questionnaire to the local culture and educational level resulted in several changes to the questionnaire. The final questionnaire was translated to Kiswahili. Interviewers underwent three days of training about the trial and how to administer the questionnaire. Questionnaires were administered in the same classroom where the information session took place and each interview lasted about 15 min.

### Treatment allocation

The assignment of caregivers into one of four groups was accomplished in two steps: first, they were divided into receiving the pamphlet (their child/children took the pamphlet home) or not, and second they were divided into receiving the information session before or after answering the questionnaire (Fig. 1). The day before the start of the information sessions we randomly assigned 400 children to receive the pamphlet or not. However, of the caregivers who showed up for an information session, the number of children who were found infected with hookworm were not sufficient for our clinical trial. To reach our target sample size (180 children with hookworm infections) we handed out more pamphlets and invited more caregivers for information sessions. In this second round of pamphlet distribution we used an alternate distribution (every other child received a pamphlet). The information session and interviews were held on the same day. On the day of an information session, those caregivers arriving early were immediately interviewed creating the control and pamphlet only groups. Caregivers arriving later attended the information session before being interviewed forming the information session only and the information session plus pamphlet groups. Not all caregivers of participating children responded to the questionnaire due to time constraints. Regardless of which group they belonged to, all caregivers attended an oral information session before being invited to sign the ICF.

### Sample size

This study was embedded in a clinical trial for which a minimum of 79 participants were required per treatment arm. Accounting for a loss to follow-up of 12%, the sample size of the clinical trial was 180 participants (90 per arm). In order to reach this sample size, 364 children were consented and screened for hookworm infections. Due to time restrictions, not all caregivers who attended an information session and provided consent could be interviewed. A mean of 62 participants per group were included in this study. In the control group, caregivers were assumed to have a 25% chance of answering correctly each of the multiple choice questions. Our sample size would enable the detection of a 20% increase in correctly answered question (from 25 to 45%) to be detected with 80% power.

### Statistical analysis

All caregivers who responded to the questionnaire were included in the analysis. The effects of the pamphlet and information session on the proportion of each question that was correctly answered were estimated using a logistic regression model adjusting for interviewer, caregiver’s age and asset index. We adjusted the *p*-values for multiple comparisons using the Benjamini-Hochberg procedure; this was done separately for pamphlet and information session [16]. Additionally, we summarize binary data using proportions.

We took into account the clustering of children within classes using a random effect. An interaction between the effects of pamphlet and information session was tested and no evidence of a synergistic effect for any of the questions was found resulting in removal of the interaction from the model.

The caregivers’ age was split into three categories: ≤ 25, 26 to 50, and ≥ 51 years. An asset index was used as a proxy of socioeconomic status. This index was generated by summing the number of assets owned by the child’s household [17]. Children with incomplete questionnaires (*n* = 5) could not be attributed an asset score.

## Results

A total of 254 caregivers responded to the questionnaire over 12 information sessions: 65 in the control group, 63 in the pamphlet only group, 62 in the information session only group, and 64 in the pamphlet plus information session group (Fig. 1).

Asset ownership information was obtained for 249 participants. Children reported to drink water from different water sources: from a well (92%), from a tap (7%), or both (1%). Only 40% of children reported to have electricity. The remaining results on asset possession are presented as an Additional file (see Additional file 4).

The pamphlet did not significantly increase caregivers’ understanding. Even though most parents assigned to receive the pamphlet reported to have indeed received it (77%, average of both groups) and read it (80% of the 97 caregivers who received it), many said they did not understand it well (Table 2). Caregivers who had more than one child were assigned to more than one caregiver group, meaning some caregivers who should not have received the pamphlet did receive it. It is important to note that literacy levels were not assessed.

**Table 2:** Caregivers who reported receiving, reading and understanding the pamphlet

	Answers	Control (*n* = 65)	Pamphlet only (*n* = 63)	IS only (*n* = 62)	IS + pamphlet (*n* = 64)
Did you receive the pamphlet?	Yes	3 (5%)	50 (79%)	8 (13%)	47 (75%)
Did you read the pamphlet?	Yes	2 (3%)	40 (63.5%)	4 (7%)	38 (60%)
How well did you understand it?^a^	Not at all	0 (0%)	1 (3%)	0 (0%)	2 (5%)
	Not well	1 (50%)	9 (23%)	0 (0%)	4 (11%)
	More or less	1 (50%)	18 (46%)	2 (50%)	13 (35%)
	Well	0 (0%)	11 (28%)	2 (50%)	18 (49%)

*IS* Information session; ^a^percentage of parents who received and read the pamphlet. We are missing data on the level of understanding of two caregivers

The proportion of caregivers responding correctly to the 17 questions varied depending on the question and group, ranging from 25 to 100% (Table 3). There was no evidence of an increase in providing correct answers with the pamphlet. However, caregivers who had attended an information session before responding to the questionnaire had a higher proportion of correct answers for most of the 17 questions, with significant increases for seven questions. Questions related to the parasite itself and concerning the procedures of this study were answered correctly more often in groups attending an information session. The full data on caregivers’ answers to every question are presented in Additional file 5.

**Table 3:** Number of caregivers (%) answering correctly to each of the questions and effect (p-value) of belonging to one of the two groups which received the pamphlet or to one of the two groups which attended an information session

	Caregiver group				*P*-value for the effect of pamphlet	*P*-value for the effect of info session
	Control	Pamphlet	Info session	Info session + pamphlet		
Multiple choice questions						
What is hookworm?	30 (46%)	33 (52%)	53 (86%)	52 (81%)	0.99	0.006
Why is it bad?	24 (37%)	26 (41%)	35 (58%)	46 (72%)	0.51	0.006
Can one be treated?	52 (80%)	54 (86%)	52 (84%)	54 (86%)	0.72	0.53
Why are we doing this study?	25 (39%)	31 (49%)	35 (58%)	34 (53%)	0.99	0.13
Who decides if your child participates?	59 (91%)	57 (91%)	60 (97%)	61 (95%)	0.99	0.27
What if your child still has hookworm?	55 (85%)	44 (71%)	50 (83%)	54 (84%)	0.51	0.53
Can you give up participating?	27 (43%)	21 (33%)	19 (31%)	23 (36%)	0.72	0.31
What about payment?	52 (81%)	47 (76%)	57 (92%)	61 (97%)	0.90	0.02
Is mebendazole safe?	25 (40%)	22 (36%)	20 (33%)	19 (30%)	0.87	0.53
Who can see your child’s information?	24 (37%)	21 (33%)	32 (53%)	30 (47%)	0.82	0.02
True or false questions (What will your child have to do if he/she participates?)						
Provide several stool samples	63 (97%)	61 (97%)	62 (100%)	63 (98%)	0.72	0.76
Provide one stool sample	17 (26%)	23 (37%)	29 (47%)	24 (38%)	0.99	0.09
Provide a urine sample	16 (25%)	20 (32%)	35 (56%)	32 (50%)	0.99	0.006
Provide a small blood sample	54 (83%)	56 (89%)	60 (97%)	61 (95%)	0.72	0.02
Pay for the treatment	52 (80%)	48 (76%)	59 (95%)	57 (89%)	0.72	0.02
Tell us how he/she feels after treatment	65 (100%)	55 (87%)	60 (97%)	61 (95%)	0.51	0.53
Allow a doctor/nurse to examine him/her	63 (97%)	59 (94%)	61 (98%)	63 (98%)	0.51	0.35

The *p*-values refer to the estimated effect of providing a pamphlet, and of the information sessions. They were calculated using logistic regression with a random effect for class. The effects of pamphlet and information session were assumed to be additive: there was no evidence for any interactions between pamphlet and information session. The *p*-values have been adjusted for multiple comparison using the Benjamini-Hochberg procedure

## Discussion

Any study including human subjects should properly inform participants before obtaining informed consent. To date, few studies have investigated to what extent participants truly comprehend all the information before signing the ICF. Additionally, since it has been shown that an ICF alone is not sufficiently good at conveying information, new methods to do so are needed. Our study aimed at testing different interventions (pamphlet alone, information session alone or both) and measuring their impact on the caregivers of participants’ knowledge. We found that the information session had a positive impact on caregivers’ understanding of the clinical trial, but receiving a pamphlet did not.

The lack of effect of the pamphlet on the caregivers’ understanding could be related to the fact that although the majority of caregivers received and read it, they did not understand it. Also, caregivers in the pamphlet only group did not have the opportunity to ask questions before being interviewed, unlike the other two intervention groups. Finally, caregivers meant to receive the pamphlet may have shown it to those not meant to receive it, diluting its effect. Hence, our finding indicates that a pamphlet might not always be an appropriate communication tool. The impact of a communication tool differs among cultural settings so different methods should be tested in different regions. In many African settings oral communication is given more value than written communication, most likely due to the low literacy levels, particularly in adults [18]. Alternative forms of conveying information such as slide shows, theaters, videos and songs have been shown to be more effective than written formats [19]. It would be useful if GCP would provide guidance on the best ways to deliver information during the informed consenting process since most clinical researchers do not have formal training in social, anthropological or communication sciences. These recommendations should be tailored for different settings.

In contrast, having attended an information session increased the proportion of correct answers given to most questions and these increases were significant for half of the questions. Most of these questions were either related to the parasite (hookworm) or to the trial design (procedures). For example, compared to caregivers who did not attend an information session, those who did knew significantly more often that their child did not have to provide a urine sample or pay for treatment and that they would need to provide a small blood sample (finger prick). Also, they were more knowledgeable concerning the mode of transmission of hookworm and its consequences on health. It is worth highlighting that in this study we had 12 information sessions of rather small size allowing us to increase engagement with caregivers. To minimize any confounding due to the speaker of the information session, the same person led every session and its content was standardized across sessions.

However, three important facts about our clinical trial were worryingly misunderstood by caregivers, even after an information session. First, although about half of the caregivers understood why we were conducting this clinical trial (to find the most effective regimen of mebendazole against hookworm), 30% responded it was because this drug had never been used before. Second, 53% of caregivers thought that their child cannot withdraw from the study once they had accepted to participate. Lastly, 62% of parents reported that nothing bad could happen after receiving treatment (the correct answer was that the drug may have some mild side effects such as abdominal pain or a headache). Our results are in line with those reported in a systematic review about informed consent comprehension in African research settings, which documented that only approximately half of caregivers understood the right to withdraw and the risks involved [10]. Thus, our results show that, although caregivers received these key messages during an information session, their comprehension was low. One reason could be that this format of transferring knowledge is not the most appropriate for communicating this type of information, particularly in a context where health literacy remains limited. Therefore, it is important to explore other formats of transmitting key messages of a clinical trial using pictures, videos or even theaters. Previous studies have shown that, for example, the use of videos significantly increases participants’ comprehension when compared to a standard informed consent procedure [20–22]. Another potential explanation for the poor understanding of caregivers might be related to the concept of “authority of knowledge”, i.e. caregivers may feel it is not their job to understand these issues or judge their legitimacy, consequently leaving the decision in the hands of people who truly know the subject. Therefore, it is possible that caregivers choose to trust the research staff, the teachers’ decision to support the study, the ethics committee approving the trial and the government allowing such studies to take place in their communities instead of making a real effort to understand themselves [23]. Further studies would be necessary to better understand “how much information is too much information?” in the context of a clinical trial in these settings and whether caregivers indeed feel that they do not need to truly understand the study background [24].

Additionally, some questions were answered correctly by most caregivers, regardless of which group they belonged to, i.e. having attended an information session was not the reason why they responded correctly. It seems to be known that treatment for hookworm exists, that caregivers should be the ones deciding whether their child participates in the trial or not, that the research staff will treat children still found infected at follow-up, that the child is asked for several stool samples during the study, that the child should let a doctor know how he/she is feeling after treatment, and that a child should accept a doctor’s physical examination.

Several limitations of our study are worth highlighting. First, the randomization was not conducted in a consistent manner. Although children were initially randomly assigned to receive the pamphlet or not, insufficient caregivers due to lack of adherence and insufficient children infected with hookworm, led to alternating distribution of the pamphlet in an attempt to balance both groups. Second, parents were assigned to each of the groups (with or without information session upon the interview) depending on their time of arrival to the information session classroom. Parents arriving first were immediately interviewed. However, parents who reached the study site first might have been more interested in the study and, therefore, possibly more knowledgeable. If this was the case, there could be some bias decreasing the effect of the information session. Third, because we were working in a single school, we could not avoid knowledge contamination between groups. It is likely that caregivers who attended some of the first information sessions talked to caregivers who had not yet attended an information session, sharing some of their newly acquired knowledge. This way, the effect of the information session may have become less apparent. Moreover, it would have been interesting to study the effect of the caregivers’ gender on understanding, documented in previous studies [25, 26], but gender was not recorded in our study. Likewise, checking for caregivers’ literacy levels would have been useful for the interpretation of our particular results. Finally, it has been shown that closed-ended questionnaires assessing knowledge tend to overestimate the participants’ understanding of informed consent information. Thus, future studies could consider including either open-ended questions or allow for spontaneous answers to avoid influencing participants’ responses [27].

## Conclusions

Although caregivers already had some awareness on the disease and study procedures they gained additional knowledge during information sessions, yet not all the important messages conveyed were truly understood. Moreover, the study found that a pamphlet was not a good tool to increase people’s knowledge. Therefore, to achieve a truly informed consent of participants and/or their caregivers, better forms of delivering information need to be found.

## Supplementary information

**Additional file 1.** Information session speech.

**Additional file 2.** Pamphlet.

**Additional file 3.** Questionnaire.

**Additional file 4.** Characteristics of participants by group (only includes participants with asset data).

**Additional file 5.** Number of parents choosing each of the responses to each question by caregiver group.

## Data Availability

All data generated and analysed during this study are included in this published article and in its additional files.

## Abbreviations

EKNZ: Ethics Committee of Northern and Central Switzerland
GCP: Good Clinical Practice
ICF: informed consent form
ICH GCP: International Conference on Harmonisation Good Clinical Practice
IS: Information session
ZAMREC: Zanzibar Medical Research and Ethical Committee
