# Supplementary material for: Informed consent procedure in a double blind randomized anthelminthic trial on Pemba Island, Tanzania: do pamphlet and information session increase caregivers knowledge?
Source: BMC Med Ethics. 2020 Jan 6;21:1. doi: 10.1186/s12910-019-0441-3 (PMC6945786; doi:10.1186/s12910-019-0441-3)
Supplement: Supplementary file 1 — Additional file 1. Information session speech. [file 12910_2019_441_MOESM1_ESM.docx]

**Additional file 1.** Information session speech

After all caregivers arrived and were seated, one of the fieldworkers went through the following points in Kiswahili:

- We are here today to talk to you about a research we are doing.
- We will explain the whole study and you can ask all the questions you want to.
- Have you ever heard of belly worms that can infect us and our children?
- Some infect us when we drink dirty water or swim in a river, for example. However, that is not the case of the type of belly worm we are going to talk to you about today.
- Our research is about one of these worms and it is called HOOKWORM (*write hookworm on the board*).
- Hookworm is a worm which is in the soil. If a person walks without shoes and steps on it, the worm can go into the foot through the skin. Then the worm goes through the blood all the way to the belly where it will live.
- Many children in Pemba have these worms in their belly. Your child could have it.
- These worms can cause many problems. For example:
- Your child may not grow well
- Your child may not be able to concentrate in school
- Because of this, he may not be able to work well when he is grown up
- There are some drugs that can kill the hookworm that live in children’s bellies.
- In our research, we want to test one drug that kills this worm. This medicine is called MEBENDAZOLE (*write it on the board and show the tablet*).
- Mebendazole is a drug that has been used for almost 50 years so we know it is safe. Sometimes it can cause a belly ache, dizziness or headache but it does not cause anything dangerous.
- What we want to check in this study is what is the amount of this medicine that kills more hookworms in children’s bellies.
- In our study we will treat 180 children who have hookworm.
- You will decide yourself if you want your child to participate. It is not us, or the doctors, or the teachers who decide – it is the parents of the child.
- If you decide your child should participate in this study and receive treatment he will have to do several things:

1. He will have to give us 2 stool samples so we can find out if he has hookworm in his belly or not.
2. He will have to let a doctor and a nurse check his health.
3. He will have to give us a small sample of finger blood to check if he has anemia (lack of iron in the blood).
4. He will receive the medicine called mebendazole. Your child will receive the treatment for 3 mornings and 3 evenings so for 3 days we will need your child to be in school all day. We will bring biscuits, water and lunch for your child. It is VERY important that your child comes the 3 days, otherwise the drug will not kill the worms in his belly like it should.
5. Two weeks after he is treated we will ask him for 2 more stool samples to check if the treatment killed the worms or if he still has hookworm in his belly.

- These are the things we will ask from your child. Any questions?
- If you want your child to participate in our research and receive the treatment, we will ask you to sign the paper you have with you – but not yet. We will help you sign it on your way out.
- If now you decide you want your child to participate but then you change your mind that is totally fine. You can give up being in the study at any moment without any consequences. Your child will still receive treatment.
- It could happen that mebendazole does not kill the hookworm in your child. If this happens, we will give him another drug called albendazole.
- It is important to mention this treatment is for free – you do not have to pay anything. We also do not pay you if your child participates but we will give you 5,000 Tanzanian Shillings to pay for your transport to the school today.
- Also, the research team members are the only people who can see your child’s personal information. We will not show it to anybody else.
- Do you have any more questions?
