# Supplementary figures and images for: Informed consent procedure in a double blind randomized anthelminthic trial on Pemba Island, Tanzania: do pamphlet and information session increase caregivers knowledge?
Source: BMC Med Ethics. 2020 Jan 6;21:1. doi: 10.1186/s12910-019-0441-3 (PMC6945786; doi:10.1186/s12910-019-0441-3)

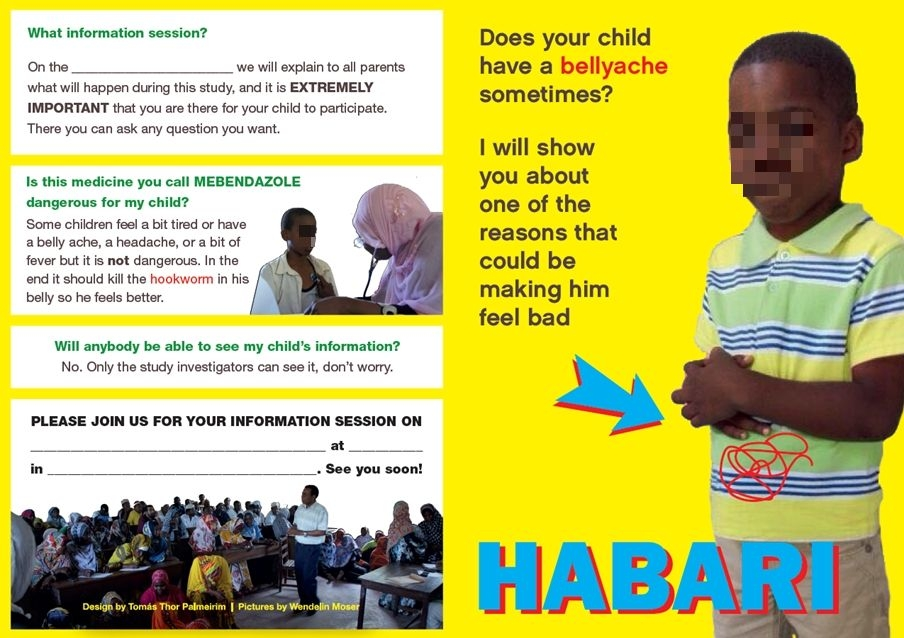


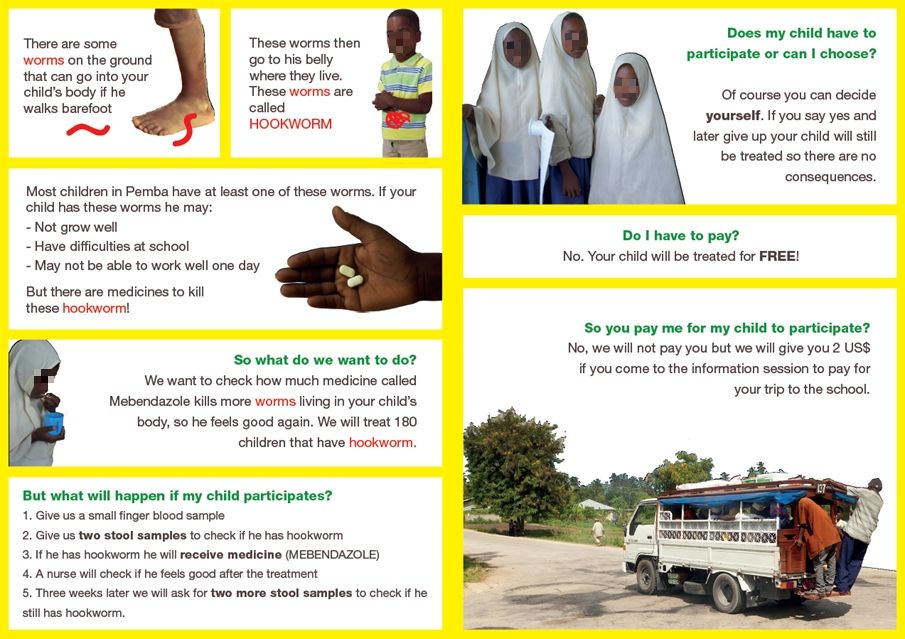

Supplement: Supplementary file 2 — Additional file 2. Pamphlet. [file 12910_2019_441_MOESM2_ESM.docx]
