# Supplementary material for: Informed consent procedure in a double blind randomized anthelminthic trial on Pemba Island, Tanzania: do pamphlet and information session increase caregivers knowledge?
Source: BMC Med Ethics. 2020 Jan 6;21:1. doi: 10.1186/s12910-019-0441-3 (PMC6945786; doi:10.1186/s12910-019-0441-3)
Supplement: Supplementary file 3 — Additional file 3. Questionnaire. [file 12910_2019_441_MOESM3_ESM.pdf]

Date: __ __ / __ __ / 2017 [DD/MM/YY] **ID** ___ ___ ___

Interviewer: ______________________________

☐ **Before** the information session ☐ **After** the information session **Total score:** [ ]

Have you ever signed an informed consent before? ☐ Yes ☐ No

If yes, do you remember what disease that study was about? ☐ Yes ☐ No

If yes, which one? ______________________________

Did your child give you this information paper we gave to him/her in school? ☐ Yes ☐ No

If yes, did you read it? ☐ Yes ☐ No

If yes, did you understand it: ☐ Not at all ☐ Not very well ☐ More or less ☐ Very well **?**

A. What is hookworm? ***Is hookworm…*** A [ ]

1) A worm that can infect us if we drink dirty water? ☐ Yes ☐ No
2) A worm that can go into our feet if we walk barefoot? ☐ Yes ☐ No
3) A worm that can infect us if we eat rotten food? ☐ Yes ☐ No
4) A worm that can get to our food through flies? ☐ Yes ☐ No
5) Don’t know.

B. Why is hookworm bad for your child? ***Is hookworm bad for your child because…*** B [ ]

1) My child will get pimples all over the body? ☐ Yes ☐ No
2) My child’s urine will become red (blood)? ☐ Yes ☐ No
3) My child may not grow well and may have difficulties at school? ☐ Yes ☐ No
4) My child will be very hungry all the time? ☐ Yes ☐ No
5) Don’t know.

C. Is it possible to treat hookworm? C [ ]

1) No, it is not possible to treat hookworm. ☐ True ☐ False
2) Yes, it is possible to treat if he eats a lot of healthy food like vegetables and fruit. ☐ True ☐ False
3) Yes, I should take him to the traditional healer. ☐ True ☐ False
4) Yes, he can receive medication that will kill the hookworm. ☐ True ☐ False
5) Don’t know.

D. What do we want to do in this study? D [ ]

1) To see if mebendazole kills the worms in your child’s belly because this drug has never been used before. ☐ True ☐ False
2) To see if mebendazole kills the worms that are in your child’s feet. ☐ True ☐ False
3) We want to see if mebendazole is better than another drug called albendazole at killing the worms. ☐ True ☐ False
4) We want to find out what is the best amount of a mebendazole to kill the hookworm in your child’s belly. ☐ True ☐ False
5) Don’t know.

E. Who <u>should</u> decide if your child should participate in this study? ***Should…*** E [ ]

1) The teacher decide? ☐ Yes ☐ No
2) Mother/Father decide? ☐ Yes ☐ No
3) The doctors or nurses decide? ☐ Yes ☐ No
4) Neighbour or relative decide? ☐ Yes ☐ No
5) Don’t know.

F. What does your child need to do if he/she participates in this study? ***Will he have to…***
(Cross all those which are correct) F ☐

1) Give us several stool samples? ☐ Yes ☐ No
2) Pay for the medication to kill the worms (mebendazole)? ☐ Yes ☐ No
3) Tell us if he is feeling well after the medication? ☐ Yes ☐ No
4) Give us a urine sample? ☐ Yes ☐ No
5) Only give us one stool sample? ☐ Yes ☐ No
6) Give us a small blood sample? ☐ Yes ☐ No
7) Accept that a doctor checks his/her health? ☐ Yes ☐ No

G. What happens if your child still has worms after the treatment? ***If he still has hookworm…*** G ☐

1) There is nothing that we can do? ☐ True ☐ False
2) We will give him/her an injection to kill the worms? ☐ True ☐ False
3) Your child should drink a lot of water? ☐ True ☐ False
4) We will give him/her another pill to kill the worms? ☐ True ☐ False
5) Don't know.

H. Can your child give up participating during the study? H ☐

1) Yes he can give up and there is no consequence. He/she will still receive treatment. ☐ True ☐ False
2) Yes but he/she will not receive treatment. ☐ True ☐ False
3) No, he cannot give up if I decide he participates he has to stay until the end of the study. ☐ True ☐ False
4) Only if the doctor and teacher agree that he can give up. ☐ True ☐ False
5) Don't know.

I. What about payment? I ☐

1) There are some costs for you: you will have to pay for your child's treatment. ☐ True ☐ False
2) There are no costs for you: the treatment is free and you will get 2$ if you came to the information session. ☐ True ☐ False
3) You will receive money if your child accepts the treatment. ☐ True ☐ False
4) You will only get money if the treatment kills the worms. ☐ True ☐ False
5) Don't know.

J. Is the treatment we will give to your child (mebendazole) safe? J ☐

1) Nothing bad can happen if he takes the treatment. ☐ Yes ☐ No
2) If your child takes the treatment he will not be able to walk for a few days. ☐ Yes ☐ No
3) If he takes the treatment, your child may feel some things like a belly ache or a headache but nothing very dangerous. ☐ Yes ☐ No
4) If your child takes the treatment he will sleep all day. ☐ Yes ☐ No
5) Don't know.

K. Who will be able to see your child's personal information? K ☐

1) Will your neighbors see it? ☐ Yes ☐ No
2) Will only study investigators see it? ☐ Yes ☐ No
3) Will your child's teacher see it? ☐ Yes ☐ No
4) Can only you see it? ☐ Yes ☐ No
5) Don't know.

Socioeconomic questions – ask the child when collecting stool

L. Which of the following do you have in your household (your mother and father own)? (cross if YES)

Y	N	
☐	☐	1. Radio
☐	☐	2. Television
☐	☐	3. Soap
☐	☐	4. Computer
☐	☐	5. Refrigerator
☐	☐	6. Fan
☐	☐	7. Bike
☐	☐	8. Motorcycle
☐	☐	9. Car
☐	☐	10. Tractor

Y	N	
☐	☐	11. Latrine
		o 11.1. Inside latrine
		o 11.2. Outside latrine
		o 11.3. Don't have.
☐	☐	12. Cell phone
☐	☐	13. Electricity
☐	☐	14. Cable (TV)
☐	☐	15. Box receiver
☐	☐	16. Satellite dish

M. Where do you get drinking water?

Y	N	
☐	☐	1. Well
☐	☐	2. Tap
☐	☐	3. Lake
☐	☐	4. Other: ____________________

N. What is the roof of your house made of?

Y	N	
☐	☐	1. Palms
☐	☐	2. Aluminum
☐	☐	3. Other: ____________________

O. What are the walls of your house made of?

Y	N	
☐	☐	1. Only clay
☐	☐	2. Clay with stones
☐	☐	3. Bricks
☐	☐	4. Other: ____________________

P. What is the type of floor in your house?

Y	N	
☐	☐	1. Soil
☐	☐	2. Clay
☐	☐	3. Cement
☐	☐	4. Tiles
☐	☐	5. Other: ____________________
