# Supplementary material for: Informed consent procedure in a double blind randomized anthelminthic trial on Pemba Island, Tanzania: do pamphlet and information session increase caregivers knowledge?
Source: BMC Med Ethics. 2020 Jan 6;21:1. doi: 10.1186/s12910-019-0441-3 (PMC6945786; doi:10.1186/s12910-019-0441-3)
Supplement: Supplementary file 4 — Additional file 4. Characteristics of participants by group (only includes participants with asset data). [file 12910_2019_441_MOESM4_ESM.docx]

**Additional file 4.** Characteristics of participants by group (only includes participants with asset data).

|  | | **No info**  **(n=64)** | **Pamphlet**  **(n=60)** | **IS**  **(n=61)** | **IS + pamphlet**  **(n=64)** | **Total** |
| --- | --- | --- | --- | --- | --- | --- |
| Age [mean (90% central range)] | | 41  (20-68) | 45  (28-68) | 42  (20-60) | 41  (20-56) | 41  (20-62) |
| Asset [n(%)] | |  |  |  |  |  |
|  | Soap | 63 (98) | 60 (100) | 61 (100) | 63 (98) | 247 (99) |
|  | Radio | 25 (39) | 33 (55) | 27 (44) | 27 (42) | 112 (45) |
|  | Television | 18 (28) | 20 (33) | 22 (36) | 20 (31) | 80 (32) |
|  | Cell phone | 61 (95) | 59 (98) | 59 (97) | 61 (95) | 240 (96) |
|  | Computer | 2 (3) | 9 (15) | 1 (2) | 3 (5) | 15 (6) |
|  | Fan | 8 (13) | 12 (20) | 10 (16) | 14 (22) | 44 (18) |
|  | Refrigerator | 10 (16) | 15 (25) | 17 (28) | 18 (28) | 60 (24) |
|  | Bicycle | 45 (70) | 38 (63) | 44 (72) | 44 (69) | 171 (69) |
|  | Scooter | 5 (8) | 15 (25) | 7 (12) | 14 (22) | 41 (17) |
|  | Car | 1 (2) | 0 (0) | 0 (0) | 2 (3) | 3 (1%) |
|  | Tractor | 0 (0) | 0 (0) | 0 (0) | 0 (0) | 0 (0) |
|  | Electricity | 22 (34) | 23 (38) | 27 (44) | 28 (44) | 100 (40) |

Note: no age was recorded for 11 caregivers and no asset data were recorded for five caregivers.
