# Supplementary material for: Informed consent procedure in a double blind randomized anthelminthic trial on Pemba Island, Tanzania: do pamphlet and information session increase caregivers knowledge?
Source: BMC Med Ethics. 2020 Jan 6;21:1. doi: 10.1186/s12910-019-0441-3 (PMC6945786; doi:10.1186/s12910-019-0441-3)
Supplement: Supplementary file 5 — Additional file 5. Number of parents choosing each of the responses to each question by caregiver group. [file 12910_2019_441_MOESM5_ESM.docx]

**Additional file 5.** Number of parents choosing each of the responses to each question by caregiver group. The correct answer is highlighted in green.

No = no information, P = only pamphlet, IS = information session, B = both pamphlet and information session

| **Question** | | **No** | **P** | **IS** | **B** |
| --- | --- | --- | --- | --- | --- |
| 1. **What is hookworm?** | | | | | |
| 1 | A worm that can infect us if we **drink dirty water** | 23 | 19 | 3 | 9 |
| 2 | A worm that can go into our feet if we **walk barefoot** | 46 | 52 | 86 | 81 |
| 3 | A worm that can infect us if we **eat rotten foot** | 11 | 8 | 7 | 3 |
| 4 | A worm that can **get to our food through flies** | 15 | 16 | 2 | 2 |
| 5 | Don’t know. | 5 | 5 | 3 | 5 |
| 1. **Why is hookworm bad for your child?** | | | | | |
| 1 | My child will get **pimples all over the body** | 34 | 10 | 8 | 5 |
| 2 | My child’s **urine will become red** (blood) | 9 | 6 | 8 | 5 |
| 3 | My child may **not grow well and may have difficulties at school** | 37 | 41 | 57 | 72 |
| 4 | My child will be **very hungry all the time** | 17 | 38 | 23 | 16 |
| 5 | Don’t know. | 3 | 5 | 3 | 3 |
| 1. **Is it possible to treat hookworm?** | | | |  |  |
| 1 | **No**, it is not possible to treat hookworm. | 5 | 3 | 5 | 3 |
| 2 | Yes, it is possible to treat **if he eats a lot of healthy food** like veg/fruit. | 15 | 10 | 10 | 8 |
| 3 | Yes, I should take him to the **traditional healer**. | 0 | 2 | 2 | 2 |
| 4 | Yes, he can receive **medication** that will kill the hookworm. | 80 | 86 | 84 | 86 |
| 5 | Don’t know. | 0 | 0 | 0 | 2 |
| 1. **What do we want to do in this study?** | | | | | |
| 1 | To see if mebendazole kills the worms in your child’s belly because this drug has never been used before. | 33 | 41 | 32 | 27 |
| 2 | To see if mebendazole kills the worms that are in your child’s feet. | 9 | 2 | 5 | 9 |
| 3 | We want to see if mebendazole is better than another drug called albendazole at killing the worms. | 11 | 6 | 3 | 11 |
| 4 | We want to find out what is the best amount of a mebendazole to kill the hookworm in your child’s belly. | 39 | 49 | 58 | 53 |
| 5 | Don’t know. | 8 | 2 | 2 | 0 |
| 1. **Who should decide if your child should participate in this study?** | | | | | |
| 1 | Teacher | 3 | 5 | 2 | 0 |
| 2 | Mother/father | 91 | 91 | 97 | 95 |
| 3 | Doctors or nurses | 6 | 5 | 2 | 5 |
| 4 | Neighbor or relative | 0 | 0 | 0 | 0 |
| 5 | Don’t know. | 0 | 0 | 0 | 0 |
| 1. **What happens if your child still has worms after the treatment?** | | | | | |
| 1 | There is **nothing that we can do** | 5 | 11 | 5 | 8 |
| 2 | We will give him/her an **injection** to kill the worms | 6 | 5 | 7 | 5 |
| 3 | Your child should **drink a lot of water** | 5 | 11 | 3 | 3 |
| 4 | We will give him/her **another pill** to kill the worms | 85 | 71 | 83 | 84 |
| 5 | Don’t know. | 0 | 2 | 2 | 0 |
| 1. **Can your child give up participating during the study?** | | | | | |
| 1 | **Yes he can give up and there is no consequence**. He/she will still **receive treatment**. | 43 | 33 | 31 | 36 |
| 2 | Yes but he/she **will not receive treatment**. | 6 | 11 | 6 | 6 |
| 3 | **No**, he cannot give up if I decide he participates he has to stay until the end of the study. | 38 | 44 | 53 | 53 |
| 4 | **Only if the doctor and teacher agree** that he can give up. | 13 | 10 | 6 | 3 |
| 5 | Don’t know. | 0 | 2 | 3 | 2 |
| 1. **What about payment?** | | | | | |
| 1 | There are some costs for you: you will have to **pay for your child’s treatment**. | 11 | 6 | 5 | 3 |
| 2 | There are **no costs for you**: the treatment is free and you will get **2$ if you came to the information session**. | 81 | 76 | 92 | 97 |
| 3 | **You will receive money** if your child accepts the treatment | 5 | 6 | 0 | 0 |
| 4 | You will **only get money if the treatment kills the worms** | 2 | 6 | 0 | 0 |
| 5 | Don’t know. | 2 | 5 | 3 | 0 |
| 1. **Is the treatment we will give to your child (mebendazole) safe?** | | | | | |
| 1 | **Nothing bad can happen** if he takes the treatment. | 44 | 57 | 61 | 62 |
| 2 | If your child takes the treatment he will **not be able to walk** for a few days. | 0 | 0 | 0 | 0 |
| 3 | If he takes the treatment, your child **may feel some things like a belly ache or a headache but nothing very dangerous.** | 40 | 36 | 33 | 30 |
| 4 | If your child takes the treatment **he will sleep all day**. | 11 | 3 | 3 | 8 |
| 5 | Don’t know. | 5 | 3 | 3 | 0 |
| 1. **Who will be able to see your child’s personal information?** | | | | | |
| 1 | Neighbors | 0 | 0 | 0 | 0 |
| 2 | Only study investigators | 37 | 33 | 53 | 47 |
| 3 | Teacher | 6 | 3 | 2 | 0 |
| 4 | Only you | 57 | 62 | 46 | 53 |
| 5 | Don’t know. | 0 | 2 | 0 | 0 |
